# Comorbidities and correlates of conduct disorder among male juvenile detainees in South Korea

**DOI:** 10.1186/s13034-017-0182-3

**Published:** 2017-09-15

**Authors:** Bum-Sung Choi, Johanna Inhyang Kim, Bung-Nyun Kim, Bongseog Kim

**Affiliations:** 1 Department of Psychiatry, Medical Research Institute, Pusan National University Yangsan Hospital, 20 Geumo-ro, Yangsan, Mulgeum-eup 50612 Republic of Korea; 2 Division of Child and Adolescent Psychiatry, Department of Psychiatry, Seoul National University College of Medicine, 101 Daehak-no, Chongno-gu, Seoul, 03080 Republic of Korea; 3 Department of Psychiatry, Sanggye Paik Hospital, Inje University College of Medicine, 1342 Dong-il Street, Seoul, 01757 Republic of Korea

## Abstract

**Background:**

The purpose of this study was to examine the rate and distribution of comorbidities, severity of childhood maltreatment, and clinical characteristics of adolescents with conduct disorder detained in a juvenile detention center in South Korea.

**Methods:**

In total, 173 juvenile detainees were recruited. We analyzed the distribution of psychiatric disorders among the sample and compared the rate of comorbidities between groups with and without conduct disorder. We compared the two groups in terms of demographic and clinical characteristics, as well as severity of childhood maltreatment and psychiatric problems, using the Young Self Report (YSR) scale.

**Results:**

A total of 95 (55%) of the detainees were diagnosed with conduct disorder, and 93 (96.9%) of them had at least one comorbid axis I psychiatric disorder. Detainees with conduct disorder had a higher number of comorbid psychiatric disorders; a higher rate of violent crime perpetration; had suffered more physical, emotional, and sexual abuse; and showed higher total YSR scores and externalizing behavior, somatic complaints, rule-breaking behavior, and aggressive behavior YSR subscale scores.

**Conclusions:**

Conduct disorder is a common psychiatric disorder among juvenile detainees in South Korea, who tend to commit more violent crimes and show more psychopathology than detainees who do not have conduct disorder. These findings highlight the importance of diagnosing and intervening in conduct disorder within the juvenile detention system.

## Background

Juvenile offenders constitute 5.1% of all criminal offenders in South Korea. Approximately 8272 juvenile offenders are newly detained in juvenile detention centers every year [1]. Previous studies reported that 40–90% of juvenile offenders had at least one psychiatric disorder [2–6], which represents an approximately three- to fourfold higher prevalence of psychiatric illness compared with the general population [7–9]. The prevalence of different psychiatric disorders varies by study; in a metaregression analysis of 13,778 boys and 2972 girls, 3.9–7.3% of the boys had major depression, 4.1–19.2% had attention deficit hyperactivity disorder (ADHD), and 40.9–64.7% had conduct disorder. Among the girls, 21.9–36.5% had major depression, 9.3–27.7% had ADHD, and 32.4–73.2% had conduct disorder [10].

Despite the high rate of psychiatric illnesses among juvenile offenders, research on the psychiatric health of this population in Asian countries, including South Korea, is limited. Park et al. [1] reported that, among 1700 inmates of three prisons, 28.1% were classified as being at high risk for depression, 33.6% had suicidal ideation, and 39.1% were diagnosed with alcohol abuse. Another study reported higher rates of depression, paranoia, antisociality, and Minnesota Multiphasic Personality Inventory (MMPI) scale hypomania among 1155 juvenile offenders compared to the general population [11]. Both studies used self-rated questionnaires, and only the latter targeted a juvenile population. To our knowledge, no South Korean study has estimated the prevalence of psychiatric disorders among juvenile offenders using Diagnostic and Statistical Manual for Mental Disorders (DSM) or International Classification of Diseases (ICD)-based criteria.

Conduct disorder is one of the most common psychiatric disorders among juvenile offenders, with the prevalence ranging from 31 to 77% [12, 13]. In previous studies, conduct disorder showed high comorbidity with substance use disorders and ADHD; all of these disorders are risk factors for higher psychiatric disorders.

The purpose of this study was to investigate the prevalence of psychiatric disorders among juvenile detainees in South Korea, and to assess patterns of comorbidity and psychopathology among those with conduct disorder.

## Methods

### Participants and procedure

In total, 200 detainees who were sentenced to 6 or 12-month detainment in a single male juvenile detention center in Seoul, South Korea, were recruited from December 2015 to January 2016. A total of 27 detainees over the age of 19 were excluded from the study, giving 173 participants. Subjects were eligible for inclusion in the study regardless of psychiatric diagnosis, degree of drug or alcohol intoxication, or fitness to stand trial. Exclusion criteria included refusal or inability to cooperate or understand the study procedures. Written informed consent was obtained from the participants after the study procedures were explained. This study protocol was approved by the Institutional Review Board of Sanggye Paik Hospital (IRB No. SGPAIK 2015-06-022-002).

Psychiatric diagnoses were confirmed using the Mini-International Neuropsychiatric Interview (MINI), which is a short, structured psychiatric interview that can detect a wide range of DSM-IV and ICD-10 psychiatric disorders [14]. The MINI has been applied for the assessment of psychiatric disorders in various criminal justice settings [15, 16]. The Korean version has well-established validity and reliability [17]. In cases of disorders not covered by the MINI, the Kiddie-Schedule for affective disorders and Schizophrenia-Present and Lifetime Version-Korean Version (K-SADS-PL-K) were used; the reliability and validity of the K-SADS-PL-K have been confirmed [18]. Diagnoses of ADHD, ODD, CD, and tic disorders were based on the behavioral disorder supplement of the K-SADS-PL-K.

The presence and degree of childhood maltreatment were evaluated using the Korean version of the Childhood Trauma Questionnaire (CTQ) [19], which has good validity and reliability [20]. The CTQ consists of 28 items; each item is rated on a five-point Likert scale and higher scores indicate more severe childhood maltreatment. The results are presented as total scores, and as scores on each of five subscales (emotional neglect, emotional abuse, physical neglect, physical abuse, and sexual abuse). We applied a moderate-to-severe cut-off score for each subscale [21, 22], and individuals who exceeded the cut-off score were categorized as juvenile detainees with a history of childhood maltreatment.

Various psychiatric symptoms were screened for using the Youth Self Report (YSR) scale, which is used widely for the assessment of emotional and behavioral problems and comprises 112 items [23]. The Korean version was standardized by Oh et al. [24]. All subscale scores were converted into T-scores, with higher scores indicating more severe symptoms. In the present study, we included the subscales of total problem behavior, internalizing, externalizing, anxiety/depression, withdrawal/depression, somatic complaints, thought problems, attention problems, rule-breaking behaviors, and aggressive behaviors.

### Statistical analysis

The demographic and clinical characteristics were compared between detainees with and without conduct disorder using independent *t*-tests for continuous variables and Chi square or Fisher’s exact test for categorical variables (such as psychiatric comorbidity status). The association between type of childhood maltreatment and conduct disorder was analyzed using logistic regression. We used multiple linear regression to evaluate the association between conduct disorder and YSR subscale scores.

All statistical analyses were performed using SPSS software (ver. 22.0; SPSS Inc., Chicago, IL, USA), and a two-tailed *p-*value <0.05 was considered significant.

## Results

The demographic and judicial characteristics of the whole sample, and of the detainees with and without conduct disorder, are presented in Table 1. The mean age was 17.5 ± 1.1 years, and all participants were male. In total, 42 (24.3%) of the participants had dropped out of school, and 104 (60.1%) were from a family with a yearly income exceeding $2500. A majority of the detainees had been living in a single parent home (n = 97, 56.1%), and 57 (32.9%) had been living with both parents; 19 (11.0%) had not been living with their parents. Property crime was the most common type of crime (n = 86, 49.7%), followed by violent crime (n = 68, 39.3%), traffic offenses (n = 42, 24.3%), and sex crimes (n = 34, 19.7%).

**Table 1:** Demographic and clinical characteristics of the detainees with and without conduct disorder

Characteristic	Whole sample (n = 173)	With conduct disorder (n = 96)	Without conduct disorder (n = 77)	p value
Age (years), mean (SD)	17.5 (1.1)	17.4 (1.2)	17.6 (1.1)	0.171
School drop out, N (%)	42 (24.3)	23 (24)	19 (24.7)	0.913
Yearly family income > $2500, N (%)	104 (60.1)	59 (61.5)	45 (58.4)	0.687
Paternal education ≥ college education, N (%)	25 (14.5)	13 (19.1)	12 (21.4)	0.750
Maternal education ≥ college education, N (%)	20 (11.6)	10 (16.7)	10 (18.2)	0.830
Living arrangements, N (%)				0.928
With both parents	57 (32.9)	31 (32.3)	26 (33.8)	
With a single parent	97 (56.1)	55 (57.3)	42 (54.5)	
No parents	19 (11.0)	10 (1.4)	9 (11.7)	
Recidivism, N (%)	154 (89)	88 (91.7)	66 (85.7)	0.213
Number of crime, mean (SD)	3.2 (1.8)	3.4 (1.9)	3.1 (1.6)	0.243
Type of crime, N (%)				
Property crime	86 (49.7)	48 (49)	40 (51.9)	0.696
Violent crime	68 (39.3)	48 (50)	20 (26)	0.001
Sex crime	34 (19.7)	14 (14.6)	20 (26.3)	0.055
Drug crime	1 (0.6)	0 (0)	1 (1.3)	0.445
Domestic violence	1 (0.6)	1 (1.0)	0 (0)	1.00
Traffic offenses	42 (24.3)	23 (24.0)	19 (24.7)	0.913
Obstruction of justice	7 (4.0)	4 (4.2)	3 (3.9)	1.00
Drunk driving	2 (1.2)	2 (2.1)	0 (0)	0.503
Others	20 (11.6)	13 (13.5)	7 (9.1)	0.363

*SD* standard deviation

There were no significant differences between the groups with versus without conduct disorder in demographic or judicial characteristics, except for a higher rate of violent crimes in the conduct disorder group (p = 0.001; Table 1).

Data on psychiatric disorder prevalence and comorbidity with conduct disorder are shown in Table 2. In total, 157 (90.8%) participants had at least one psychiatric diagnosis, and the most common axis I psychiatric disorder was alcohol use disorder (n = 100, 57.8%), followed by conduct disorder (n = 96, 55.5%), bipolar disorder (n = 82, 47.4%), and ADHD (n = 61, 35.3%). Antisocial personality traits were present in 83 (48%) detainees.

**Table 2:** Prevalence of psychiatric disorders among detainees and comorbidity with conduct disorder

Diagnosis	Whole sample (n = 173)	With conduct disorder (n = 96)	Without conduct disorder (n = 77)	p value
Any psychiatric disorder, except conduct disorder	154 (89.0)	93 (96.9)	61 (79.2)	<0.001
Number with diagnosis, N (%)				
Major depressive disorder	50 (28.9)	41 (21.9)	9 (11.7)	0.079
Bipolar disorder	82 (47.4)	59 (61.5)	23 (29.9)	<0.001
Alcohol use disorder	100 (57.8)	66 (68.8)	34 (44.2)	0.001
Substance use disorder	8 (4.6)	4 (4.2)	4 (5.2)	1.00
Schizophrenia	19 (11.0)	11 (11.5)	8 (10.4)	0.823
Eating disorder	6 (3.5)	6 (6.3)	0 (0)	0.026
ADHD	61 (35.3)	40 (41.7)	21 (27.3)	0.049
Tic disorder	47 (27.2)	24 (25.0)	23 (29.9)	0.474
ODD	14 (8.1)	0 (0)	14 (18.2)	<0.001
Antisocial personality trait	83 (48.0)	62 (64.6)	21 (27.3)	<0.001
Anxiety disorder	44 (25.4)	30 (31.3)	14 (18.2)	0.050

*AHDH* attention deficit hyperactivity disorder, *ODD* oppositional defiant disorder

In total, 96 (55.5%) detainees had a diagnosis of conduct disorder, of whom 93 (96.9%) had at least one comorbid axis I psychiatric disorder. Detainees with conduct disorder had a higher rate of comorbidity compared to those without (p < 0.001), and the most common axis I comorbid disorder was alcohol use disorder (n = 66, 68.8%), followed by bipolar disorder (n = 59, 61.5%) and ADHD (n = 40, 41.7%). All of the psychiatric disorders—except for major depressive disorder, substance use disorder, tic disorders, and anxiety disorders—were more frequently diagnosed in the conduct disorder than in the non-conduct disorder group (all p < 0.05).

The detainees with conduct disorder showed significant associations with emotional abuse [odds ratio (OR) = 1.26, 95% confidence interval (CI) 1.06–1.43; p = 0.009], sexual abuse (OR = 1.23, 95% CI 1.03–1.46; p = 0.022), and physical abuse (OR = 1.23, 95% CI 1.06–1.43; p = 0.008), and all associations remained significant after adjusting for age, living arrangements, socioeconomic status, and the presence of psychiatric comorbidities (Table 3).

**Table 3:** Association of childhood maltreatment and conduct disorder

Variables	Whole sample (n = 173	With conduct disorder (n = 96)	Without CD (n = 77)	Unadjusted OR	95% CI	p value	Adjusted OR^a^	95% CI	p value
Child maltreatment	136 (78.6)	76 (79.2)	60 (77.9)	1.019	0.849–1.223	0.843	1.01	0.82–1.24	0.942
Type of childhood maltreatment									
Emotional abuse	54 (31.2)	38 (39.6)	16 (20.8)	1.257	1.059–1.492	0.009	1.252	1.04–1.51	0.018
Sexual abuse	49 (28.3)	34 (35.4)	15 (19.5)	1227	1.029–1.462	0.022	1.209	1.00–1.46	0.048
Physical abuse	87 (50.3)	57 (59.4)	30 (39.0)	1.230	1.055–1.434	0.008	1.271	1.07–1.51	0.006
Emotional neglect	92 (53.2)	49 (51.0)	42 (55.8)	0.953	0.820–1.108	0.529	1.370	0.70–2.70	0.364
Physical neglect	93 (53.8	49 (51.0)	44 (57.1)	0.940	0.809–1.093	0.424	0.934	0.79–1.10	0.418

^a^Adjusted for age, living arrangements, SES, and presence of psychiatric disorders

Scores on YSR subscales were higher in the conduct disorder versus non-conduct disorder group, including total problem behavior (β = 1.57, 95% CI 0.47–2.67; p = 0.005), externalizing behavior (β = 2.33, 95% CI 1.27–3.40; p < 0.001), somatic complaints (β = 0.58, 95% CI 0.01–1.16; p = 0.047), rule-breaking behavior (β = 1.41, 95% CI 0.78–2.03; p < 0.001), and aggressive behavior (β = 1.15, 95% CI 0.45–1.85; p = 0.001) after adjusting for age and the presence of psychiatric comorbidities (Table 4).

**Table 4:** Association of YSR scores with conduct disorder

Variables	With conduct disorder (n = 96)	Without conduct disorder (n = 77)	β	95% CI	p value
Total problem behavior	57.2 (14.2)	49.9 (13.3)	1.57	0.47 to 2.67	0.005
Internalizing	51.6 (13.4)	46.6 (12.9)	1.034	−0.10 to 2.08	0.052
Externalizing	65.6 (13.5)	55.3 (13.5)	2.332	1.27 to 3.40	<0.001
Anxious/depressed	55.3 (7.5)	53.6 (6.3)	0.39	−0.16 to 0.95	0.166
Withdrawn/depressed	55.4 (7.3)	54.0 (6.4)	0.26	−0.29 to 0.81	0.353
Somatic complaints	56.1 (8.2)	53.7 (5.7)	0.581	0.01 to 1.16	0.047
Thought problems	56.2 (7.9)	53.8 (6.0)	0.553	−0.1 to 1.12	0.055
Attention problems	55.6 (7.5)	53.9 (8.0)	0.35	−0.27 to 0.97	0.261
Rule-breaking behavior	69.7 (7.4)	63.5 (8.6)	1.41	0.78 to 2.03	<0.001
Aggressive behavior	59.6 (10.0)	54.5 (7.1)	1.15	0.45 to 1.85	0.001

Adjusted for age and presence of psychiatric comorbidity

*YSR* the Youth Self Report scale

## Discussion

Research on the prevalence of psychiatric disorders among detained adolescents is still limited in comparison to analogous research in adults. Nevertheless, reports of psychiatric prevalence studies of adolescents have been published with increasing frequency over the past few years.

The main objectives of this study were to document the rate and distribution of comorbidities, severity of childhood maltreatment, and clinical characteristics of adolescents with conduct disorder detained in a juvenile detention center in South Korea.

Many of the juvenile offenders in our study had psychiatric disorders, including alcohol use disorder, conduct disorder, bipolar disorder, and ADHD. The percentage of detainees with at least one psychiatric axis I disorder was 90.8%, which is very high compared to the rates reported among the general adolescent population, and is in the range reported in previous studies. Alcohol abuse (57.8%) was the most common disorder, followed by conduct disorder (55.5%), bipolar disorder (47.4%), and ADHD (35.3%). Additionally, antisocial personality traits were identified in 48% of the participants. Previous studies have shown that personality disorder is highly prevalent in incarcerated juvenile populations [25]. However, a diagnosis of antisocial personality disorder is still possible above 18 years of age if there is evidence of conduct disorder with an onset prior to 15 years of age; thus the term ‘trait’ was used rather than ‘disorder’. These findings are similar to the results of Collins et al., in that the mean prevalence of any disorder was 69.9% (95% CI 69.5–70.3), with conduct disorder occurring most frequently (46.4%; 95% CI 45.6–47.3), followed by substance use disorder (45.1%; 95% CI 44.6–45.5), oppositional defiant disorder (19.8%; 95% CI 9.2–20.3), and ADHD (13.5%; 95% CI 13.2–13.9) [26]. In a meta-analysis by Fazel et al., high rates of psychotic illness (male adolescents, 3.3%), major depression (10.6%), ADHD (11.7%), and CD (male adolescents, 52.8%) were described [10]. Despite methodological differences between the two studies, overall prevalence rates for ADHD (Fazel et al., 11.7%, compared with 13.6% in our study), CD (52.8% vs. 38.8%), and major depression (10.6% vs. 10.0%) were similar [10]. As expected, conduct disorder was the most prevalent of the disorders studied, with a similar prevalence in both sexes of slightly more than 50% [10]. A report by the American Academy of Pediatrics estimated the prevalence ranges as follows: 1–6% for psychosis, up to 50% for ADHD, and 20–60% for conduct disorder [27]. Thus, the risk of conduct disorder is five to tenfold higher than that of the general population [10].

Another finding of the current study was that the rate of violent crimes among the conduct disorder group was higher than that of the non-conduct disorder group. Out of a total of 96 (55.5%) detainees who had a diagnosis of conduct disorder, 93 (96.9%) had at least one comorbid axis I psychiatric disorder. Those with conduct disorder had a higher rate of comorbidities than those without, and the most common axis I comorbid disorder was alcohol use disorder, followed by bipolar and ADHD. With the exceptions of major depressive disorder, substance use disorder, tic disorders, and anxiety disorders, all psychiatric conditions were more frequently diagnosed in the conduct disorder than in the non-conduct disorder group. One main implication arises from these findings: mental disorders are markedly more common among adolescents in detention than among age-equivalent individuals in the general population. The largest increase in risk among detainees is for conduct disorder; for male adolescent detainees, the risk of conduct disorder is five- to tenfold higher than that of the general population [10].

Regarding the YSR subscales, including total problem behavior, externalizing behavior, somatic complaints, rule-breaking behavior, and aggressive behavior, after adjusting for age and the presence of psychiatric comorbidities, scores for the conduct disorder group were consistently higher. No significant differences were found on the other subscales, including internalizing behavior, anxious/depressed behavior, withdrawn/depressed behavior, thought problems, and attention problems, after adjusting for age and the presence of psychiatric comorbidities. Additionally, Rosenblatt et al. [28] reported that juvenile offenders displayed increased functional impairment due to conduct and externalizing behavioral problems compared to the general adolescent population.

Although conduct disorder is a psychiatric condition commonly observed among juvenile detainees in South Korea, available psychiatric interventions of for this population remain limited. The present results confirm that detainees with conduct disorder had higher rates of comorbid axis I psychiatric disorders and violent crime perpetration, and had suffered more physical, emotional, and sexual abuse than those without conduct disorder. These findings suggest that the diagnosis of, and interventions for, conduct disorder within the juvenile detention system are important for the prevention of further damage to juvenile detainees.

The present study also demonstrated that detainees with conduct disorder had more severe psychopathologies than those without conduct disorder; thus, designing intervention programs will be necessary. Furthermore, additional research on the treatment of youth detainees with conduct disorder will be necessary. Subsequent studies aimed at identifying the traits of youth detainees with conduct disorder, such as callous unemotional traits, may lead to the development of more effective treatments for juvenile detainees with these characteristics.

There were some noteworthy limitations to this study. First, we included only male subjects, as the juvenile detention center from which the participants were drawn was for males only; this may limit the generalizability of the findings. Second, the detainees without conduct disorder also had high rates of psychiatric comorbidity, and there were insufficient detainees without a psychiatric disorder to act as a control group for the conduct disorder detainees. Therefore, further studies including control groups (which could be detainees without any psychiatric disorder or adolescents drawn from the general population) could help to clarify the results. Third, because we conducted the study inside the detention center, the detainees were the only informants and we were unable to obtain information from any other source. Fourth, rather than the MINI KID, the MINI was used to diagnose psychiatric disorders. The use of an adult assessment tool may be a limitation in that it does not fully cover child and adolescent psychiatric diagnoses. Finally, the detainees were drawn from a single detention center; further large-scale studies including detainees from other areas and detention centers are thus warranted.

## Conclusions

Almost all of the juvenile detainees that we recruited from a detention center in South Korea had at least one psychiatric disorder. The most common disorder was alcohol use disorder, followed by conduct disorder and antisocial personality disorder. The detainees with conduct disorder had higher rates of comorbid axis I psychiatric disorders and violent crime perpetration; had suffered more physical, emotional, and sexual abuse; and exhibited more severe psychopathology than those without conduct disorder. These findings highlight the importance of diagnosing and intervening in conduct disorder within the juvenile detention system.
